# Principles and Practice of Bandaging

**Published:** 1892-09

**Authors:** 


### Book Notices.

---

THE PRINCIPLES AND PRACTICE OF BANDAGING: By Gwilym G. Davis, M. D., Universities Pennsylvania and Gottingen. Geo. S. Davis, Publisher, Detroit, Mich. Cloth, $3 net.

"Bandaging, while not a science, is, nevertheless, governed by something more than mere empiricism; and it is one of the objects of this work to direct attention to the fundamental bandages, and of the importance of first learning principles and then their application in the form of the various special bandages."

* * * "Good results in fractures and efficacy in surgical dressings depend just as much upon the attention given to the bandaging as do the results in abdominal surgery to the manipulations employed in them." The practitioner who has neglected this important branch of study will find Dr. Davis' manual a valuable aid; all should have it for reference.

